# Global cerebral infarction after aortic arch replacement surgery in a patient with postoperatively revealed factor XII deficiency: a case report

**DOI:** 10.1186/s13256-020-02470-1

**Published:** 2020-09-11

**Authors:** Keisuke Yoshida, Shiori Tanaka, Yuki Sato, Kazuhiro Watanabe, Kenichi Muramatsu, Masahiro Murakawa

**Affiliations:** 1 Department of Anesthesiology, Aidu Chuo Hospital, 1-1, Tsuruga-machi, Aizuwakmatsu, Fukushima, 965-8611 Japan; 2 Department of Anesthesiology, Fukushima Medical University, 1, Hikariga-oka, Fukushima, Fukushima, 960-1295 Japan; 3 Department of Cardiovascular Surgery, Aidu Chuo Hospital, 1-1, Tsuruga-machi, Aizuwakmatsu, Fukushima, 965-8611 Japan

**Keywords:** Factor XII (FXII), Factor XII deficiency, Activated clotting time (ACT), Heparin, Case report

## Abstract

**Background:**

This case report presents a case of a patient with global cerebral infarction of uncertain etiology following an emergency surgery for acute type A aortic dissection. As a result, factor XII deficiency was revealed postoperatively. To date, there have been several reports of cardiovascular surgery in patients with factor XII deficiency. However, all previous reports were of patients whose factor XII deficiency had been detected preoperatively; therefore, before this, there had been no reports of complications associated with factor XII deficiency following cardiovascular surgery.

**Case presentation:**

We report a case of emergency aortic arch replacement surgery for acute type A aortic dissection in a 57-year-old Japanese man. A blood test prior to the surgery showed coagulopathy, a platelet count of 117 × 10^9^/L, a prothrombin time–international normalized ratio of 1.78, an activated partial thromboplastin time of 69.7 seconds, and fibrinogen < 50 mg/dl. A smaller-than-usual dose of heparin (8000 IU) was administered because the patient’s activated clotting time was extremely prolonged (> 999 seconds). After the heparin administration, the activated clotting time, measured every 30–60 minutes, remained unchanged (> 999 seconds); therefore, additional heparin was not administered during the surgery, and there was no clinical problem during cardiopulmonary bypass. However, a diagnosis of global cerebral infarction was made on the first postoperative day. An additional blood coagulation test performed on postoperative day 9 revealed factor XII deficiency (8.0%). Regarding the reason that global cerebral infarction occurred in the present case, two reasons were considered: One was factor XII deficiency itself, and the other was low-dose heparin administration during the cardiopulmonary bypass due to excessively prolonged activated clotting time caused by factor XII deficiency.

**Conclusions:**

Factor XII deficiency should be considered in patients with prolonged activated clotting time and spontaneous thrombosis in vascular surgeries. Moreover, the present case emphasizes that management of heparin during cardiopulmonary bypass should not be performed on the basis of activated clotting time monitoring alone, especially in a case with prolonged activated clotting time.

## Background

Although techniques and materials for anesthesia, extracorporeal circulation, and surgery have been developed, the in-hospital mortality rate of the surgical treatment of acute type A aortic dissection was reportedly 22%, and 5.9% of the deaths associated with aortic dissection were due to brain damage. Thus, aortic dissection caused brain damage. [1]. The present report describes a case of a patient with global cerebral infarction of uncertain etiology following an emergency surgery for acute type A aortic dissection. As a result, factor XII (FXII) deficiency was revealed postoperatively. Written informed consent for the publication of this article was obtained from the family of the patient.

## Case presentation

A 57-year-old Japanese man, height 170 cm, weight 75 kg, was carried to our hospital with sudden-onset chest/back pain. His only past medical history was untreated hypertension, and he had had no episode of blood coagulation abnormality. He was not taking any medication, and no significant family history was noted. When he arrived at our hospital, he was alert (Glasgow Coma Scale score 15), and he had no paralysis. Although his circulatory dynamics were almost stable, he was diagnosed with acute type A aortic dissection by contrast-enhanced computed tomography (CT). The aortic dissection extended to the abdominal aorta, left common iliac artery, and brachiocephalic artery; however, the persistence of perioperative malperfusion was not confirmed by CT. Thus, emergency aortic arch replacement surgery was scheduled. Before the surgery, there was no pericardial effusion and no major aortic valve regurgitation detected by transthoracic echocardiography, and the patient’s electrocardiogram revealed no abnormality. The results of arterial blood gas analysis were within the normal ranges, except for an increase in lactate of 4.60 mmol/L. Although the patient had no clinical bleeding tendency, a blood test prior to surgery showed coagulopathy: platelet count (PLT) 117 × 10^9^/L, prothrombin time–international normalized ratio (PT-INR) 1.78, activated partial thromboplastin time (APTT) 69.7 seconds, fibrinogen < 50 mg/dl, and D-dimer 1560 μg/ml.

General anesthesia was induced with midazolam 5 mg, fentanyl 200 μg, and rocuronium 50 mg and maintained with sevoflurane 1.5% or midazolam (during cardiopulmonary bypass [CPB]), intermittent administration of fentanyl (total 600 μg), remifentanil 0.15–0.3 μg/kg/minute, and rocuronium. During the surgery, an electrocardiogram, left radial and left dorsal pedis arterial pressure, pulse oximetry, central venous pressure, regional cerebral oxygen saturation (rSO_2_) (INVOS 5100C; Medtronic, Boulder, CO, USA), bispectral index, and transesophageal echocardiography were monitored. Left and right rSO_2_ values before the induction of anesthesia were 74% and 75%, respectively.

A smaller-than-usual dose (8000 IU) of heparin was administered because activated clotting time (ACT) prior to heparin administration was extremely prolonged (> 999 seconds). ACT was assessed by using the HMS Plus system (Medtronic). Then, the left femoral artery, right subclavian artery, left femoral vein, and superior venae cavae were cannulated to establish CPB (MERA exceline CPB circuit; SENKO MEDICAL INSTRUMENT, Tokyo, Japan). There were no clinical signs of coagulopathy during opening of the chest or cannulation.

Following establishment of CPB, the patient was cooled to 25 °C tympanic temperature. Perfusion pressure was maintained at approximately 60 mmHg during CPB. Hemiarch replacement (replacement of the ascending aorta, proximal aortic arch, and right innominate artery) was performed by using the open distal anastomosis technique under circulatory arrest with brain protection by antegrade selective cerebral perfusion. The circulatory arrest time was 49 minutes. Moreover, aortic root replacement (Bentall procedure) was performed after reestablishing CPB via the graft anastomosed to the distal end. During the surgical procedure, the rSO_2_ value was maintained between 60% and 75%. ACT assessed every 30–60 minutes after the first heparin administration was unchanged, being > 999 seconds; therefore, additional heparin was not administered during surgery. There was no clinical problem during CPB.

After the CPB, 720 ml of fresh frozen plasma (FFP) and protamine sulfate 200 mg were administered, then ACT decreased to 159 seconds. Moreover, oozing persisted after first administration of FFP and protamine; thus, 1200 ml of FFP, 800 ml of platelet concentrates (PC), fibrinogen concentrate 3 g, and tranexamic acid 2 g were needed after the CPB for hemostasis, and ACT was 137 seconds at the end of surgery. The bilateral rSO_2_ gradually decreased from the end of CPB and was approximately 40% at the end of surgery. The CPB time was 492 minutes; the operative time was 667 minutes; the anesthesia time was 739 minutes; the intraoperative urine volume was 1185 ml; and the bleeding volume was approximately 5500 ml. After the surgery, the patient was transferred to the intensive care unit (ICU) with artificial respiration.

A blood test performed soon after the surgery showed the following values: PLT 123 × 10^9^/L, PT-INR 1.52, APTT 38.3 seconds, fibrinogen 124 mg/dl, and D-dimer 116 μg/ml. After arrival in the ICU, bleeding from the drains in the pericardial cavity continued, and red blood cells and FFP were administered. On the first postoperative day (POD 1), dilation of the pupils and loss of light reflex were observed. A head CT indicated diffuse cerebral edema, and a diagnosis of global cerebral infarction was made. Although FFP and PC were administered on an as-needed basis from POD 1 to POD 7, ACT had been prolonged to approximately 150–200 seconds without using heparin. An additional blood coagulation test performed at POD 9 (Table 1) indicated FXII deficiency (8.0%). At this point, global cerebral infarction was confirmed, and a poor prognosis was expected. Therefore, with written informed consent from the family of the patient, additional intervention for FXII deficiency was not performed. The patient died on POD 14.

**Table 1:** Additional blood coagulation test at postoperative day 9

Parameter (unit)	Results	Reference values
PLT (× 10^9^/L)	72	140–379
PT-INR	1.22	0.90–1.13
APTT (seconds)	38.8	26.0–38.0
Fibrinogen (mg/dl)	779	170–410
D-dimer (μg/ml)	12.6	0.00–1.00
AT-III (%)	90	80–130
Factor II (%)	98.3	66.0–118.0
Factor V (%)	68.1	73.0–122.0
Factor VII (%)	102.4	54.0–162.0
**Factor XII (%)**	**8.0**	**36.0–152.0**
Factor VIII inhibitor (BU/ml)	< 0.5	0.00–1.00
Factor IX inhibitor (BU/ml)	< 0.5	0.00–1.00
Plasminogen activity (%)	105	80–130
Protein C activity (%)	76	70–150
Protein S activity (%)	119.2	63.5–149.0
Lupus anticoagulant	1.1	0.0–1.2
Anticardiolipin IgG antibodies (U/ml)	7	0–9

*Abbreviations: APTT* Activated partial thromboplastin time, *AT-III* Antithrombin III, *BU* Bethesda unit, *IgG* Immunoglobulin G, *PLT* Platelet count, *PT-INR* Prothrombin time - international normalized ratio

The line of Factor XII is bolded for emphasis

## Discussion and conclusions

The present case of postoperatively revealed FXII deficiency resulted in a regrettable outcome due to global cerebral infarction after aortic arch replacement surgery. FXII was first discovered as Hageman factor in 1955 [2] and is currently known as one of the contact factors that initiate the intrinsic pathway of the clotting cascade. Although FXII deficiency is associated with prolongation of APTT [3], it is often found by chance in preoperative examinations because FXII deficiency does not clinically impair hemostasis [4]. To date, there have been several reports of cardiovascular surgery in patients with FXII deficiency [5, 6]. However, all previous reports were of patients whose FXII deficiency had been detected preoperatively; therefore, there had been no reports of complications associated with FXII deficiency following cardiovascular surgery. To the best of our knowledge, this is the first report in which FXII deficiency was found postoperatively after cardiovascular surgery.

In our patient’s case, although coagulopathy had existed since before the surgery, we considered that it had been caused by disseminated intravascular coagulation (DIC). That is, the blood test prior to surgery reflected hyperfibrinolysis status at false lumen, and insufficient attention was given to APTT prolongation. However, abnormal prolongation of ACT had persisted after the surgery. Thus, several days after the surgery, we began to suspect that there was an unknown underlying blood coagulation disorder, and an additional blood coagulation test was performed at POD 9. The test revealed a severe decrease in FXII despite FFP being occasionally administered after surgery. In retrospect, if DIC had been the cause of the coagulation disorder, other coagulation factors would also have been decreased. Hence, the possibility of FXII deficiency was extremely high in our patient’s case, although genetic testing for definitive diagnosis was not performed.

ACT is commonly used for monitoring heparin in cardiovascular surgery. FXII is the first factor in the intrinsic coagulation pathway. FXII is activated when bound to nonphysiological surfaces [4, 7]. Thus, ACT dependent on contact activation is prolonged in FXII deficiency [4]. Thrombocytopenia and antiphospholipid antibody syndrome have also been reported to be factors that cause ACT prolongation [8]. In our patient’s case, postoperative PLT was approximately 100 × 10^9^/L, and results of tests for lupus anticoagulant and anticardiolipin antibodies were negative; therefore, the prolongation of ACT was not due to these factors.

Patients with acute type A aortic dissection are commonly found to have coagulation disorder preoperatively, such as by low PLT and fibrinogen and high D-dimer and PT-INR [9]. However, it is impractical to conduct advanced coagulation tests in all cases prior to emergency surgery, because FXII deficiency is extremely infrequent (1 in 1 million individuals) [10]. To prevent outcomes such as the one in our patient’s case, heparin concentrations should be closely monitored simultaneously with ACT measurement during CPB [6, 11]. Although their patients had been diagnosed with FXII deficiency preoperatively, Uppal *et al.* [11] reported that heparin concentration monitoring allowed safe heparin anticoagulation in patients with FXII deficiency. If coagulation abnormalities are suspected, advanced coagulation tests, which we did not perform in our patient’s case because of the difficulty of emergency advanced coagulation tests during nighttime in our hospital, should be performed in parallel with the surgery. In addition, coagulation management using viscoelastic testing such as thromboelastography would be effective in such cases [6, 12], although it is not available in our hospital. Moreover, a chromogenic anti-FXa assay might be useful for the monitoring of high-dose heparin in contact pathway deficiencies such as FXII deficiency [13]. There have been several reports of surgery being successfully completed by adding FFP before surgery in patients with preoperatively diagnosed FXII deficiency [5, 14]. Therefore, administration of FFP may be an option when FXII deficiency is suspected. Our hospital mainly used ACT for heparin management during CPB; however, we have started monitoring heparin concentration as well as ACT since this experience.

Regarding the reason why global cerebral infarction occurred in the present case, two mechanisms are considered. The first is FXII deficiency itself. FXII is involved in both coagulation and fibrinolysis: Activated FXII activates not only FXI but also plasma kallikrein, high-molecular-weight kininogen, and bradykinin (kallikrein–kinin system). The plasma kallikrein promotes fibrinolysis; that is, the contact system contributes to procoagulant disorders [4]. FXII can cause embolism [15], and it can result in clotting of the implanted bypass grafts [16]. The second is that medical staff hesitate to perform adequate anticoagulation therapy due to extremely prolonged coagulation tests in patients with FXII deficiency. In the present case, the dose of heparin administered during the CPB was low because of excessively prolonged ACT due to the involvement of FXII deficiency. At the time the surgery started, the cause of overprolonged ACT was unknown, so the initial dose of heparin was decided by anesthesiologists, cardiovascular surgeons, and perfusionists on the basis of our limited experience. As a result, a microthrombus that formed in the CPB circuit caused scattered embolic strokes, and they might have contributed to global cerebral infarction. Symptoms of cerebral embolism were prominent after surgery, suggesting that a microthrombus had already been formed and embolized to capillary vessels in the brain during antegrade selective cerebral perfusion. This theory is also supported by the findings that the rSO_2_ gradually declined after the CPB and a major cerebral vasculature embolism was not seen on postoperative head CT. Moreover, our patient needed a relatively large amount of FFP, PC, and fibrinogen for hemostasis after CPB. Although detailed coagulation tests were not performed intraoperatively in the present case, administration of these blood products was unavoidable on the basis of clinical findings. It is difficult to rule out the slight possibility that the use of these FFP, PC, and fibrinogen was associated with the patient’s postoperative cerebral infarction. However, blood tests after the surgery indicated that supplementation of these coagulation factors was minimal. Therefore, the effect of blood products on the postoperative cerebral infarction was negligible.

Heparin-induced thrombocytopenia (HIT) is well known as a cause of systemic thrombosis in the perioperative period of cardiovascular surgery. There are two types of HIT: type 1 (direct interaction between heparin and platelets) and type 2 (antibody mediated) [17]. In the present case, tests for diagnosing HIT were not performed. The onset of HIT type 1 is 1–4 days, but the symptom of HIT type 1 is mild, and thromboembolic sequelae are none [17]. Although HIT type 2 exhibits mild thrombocytopenia as in the present case, thrombocytopenia and thrombosis related to HIT type 2 usually occur 5–14 days after initiation of heparin therapy [17, 18]; thus, two types of HIT were ruled out in the present case.

In conclusion, FXII deficiency should be considered in patients with prolonged ACT and spontaneous thrombosis in vascular surgeries. Moreover, the present case emphasizes that ACT is not a reliable marker for coagulation status in FXII deficiency. Hence, management of heparin during CPB should not be performed on the basis of ACT monitoring alone, but it is necessary to make a comprehensive judgment using multiple monitoring strategies, especially in cases with prolonged ACT.

## Data Availability

Not applicable.

## Abbreviations

ACT: Activated clotting time
APTT: Activated partial thromboplastin time
CPB: Cardiopulmonary bypass
CT: Computed tomography
DIC: Disseminated intravascular coagulation
FFP: Fresh frozen plasma
FXII: Factor XII
HIT: Heparin-induced thrombocytopenia
ICU: Intensive care unit
PC: Platelet concentrates
PLT: Platelet count
POD: Postoperative day
PT-INR: Prothrombin time–international normalized ratio
rSO_2_: Regional cerebral oxygen saturation
